# The Use of Ipecacuanha in Certain Stomach Disorders

**Published:** 1854-09

**Authors:** 

**Affiliations:** Physician to King’s College Hospital


The Use of Ipecacuanha in certain Stomach Disorders.—By Dr. Budd, Physician to King's College Hospital.—Ipecacuanha increases the secretions of the stomach in a greater degree, probably, than any other medicine we possess. It increases, as is well known, the secretion of the skin, and the secretion of the mucous membrane of the air-tubes; but it increases in much greater degree, the secretion of the mucous membrane of the stomach, to which it is directly applied. An emetic dose of ipecacuanha causes a copious secretion of mucous and of gastric acid, which is rejected by vomiting. Doses too small to excite vomiting or nausea increase the secretion of the gastric juice, and in so doing render digestion quicker and stronger. Its right application, therefore, is where digestion is slow, or where, through slow and feeble digestion, nettlerash or other secondary disorders are bred.

Small doses of ipecacuanha were first recommended as a remedy in indigestion in a tract published in 1785, by a French naturalist, M. Daubenton, well known from the aid he afforded Buffon in the production of his splendid work on natural history. Daubenton was educated for medicine, but left the practice of it for his favorite study of natural history. In the tract in which he recommends small doses of ipecacuanha in indigestion, he says: "I have repeatedly experienced beneficial effects from it in my own person that surpasses my expectations, and I have prescribed it to many others, with whom it has had similar success. I consider it, therefore, a duty to publish the observations on the utility of this simple remedy, for the benefit of those persons who have delicate stomachs, and as particularly useful in that form of indigestion which is so frequently found to attend the turn of life."

Daubenton is careful in stating that the cases in which ipecacuanha is useful are where digestion is slow,—where the food lies heavy on the stomach, and there is an inability for mental or bodily exertion for some time after meals,—a kind of disorder which is, he states, especially common in men of middle age, or beyond it, who lead sedentary lives.

He believed that ipecacuanha owes its efficacy in such cases to its exciting peristaltic action in the stomach, and imparting an energy to its glands.

He recommends that it should be given in the morning fasting, and in quantity sufficient to occasion a slight feeling of vermiculating motion in the stomach, but without causing any sensation of pain or nausea. The quantity requisite to produce this effect varies, he says, in different persons from a quarter of a grain to two grains. He advises, therefore, that very small doses be given at first, which may be gradually increased till a sensible effect is produced.

In the beginning of this century an English translation of Daubenton's tract was published by Dr. H. P. Buchan, and rapidly sold.

In the preface to this translation, Dr. Buchan says:—"The translator of this little tract can truly declare, that since he became acquainted with the information contained in it, his practice in the complaints here enumerated have been more successful and satisfactory than it was previously, and his sole motive for publishing the translation, which was originally made for his own private use, is to extend the knowledge of what he conceives to be a practical improvement in the art of medicine."

The medicine in this application being thus introduced to the profession in this country, was much employed for a time, and then fell into disuse, in consequence, the author believes, of its having been employed indiscriminately in various kinds of indigestion, and often, therefore, in kinds to which it is not suited, and which it would tend to aggravate rather than remedy.

It is clearly impossible that any medicine having a definite mode of action—whether it be to increase secretion or to restrain it—can be used successfully in stomach disorders, unless the various kinds of stomach disorders be distinguished, and the medicine be given only in that kind, or in those kinds, to which it is suited.

Here, as in other departments of medicine, we must rightly distinguish kindred disorders before we can learn the right use and the power of remedies.

The author has used ipecacuanha as a remedy for indigestion for several years, and he believes that there is no other medicine we know of so effectual in removing the uneasiness and sense of oppression after meals, and the various other evils that result from slow digestion.

Small doses of rhubarb, ginger, and pepper, have a similar kind of action, and may be given singly or together for the same pu pose. The author generally prescribes ipecacuanha, from half grain to a grain, in a pill, with three or four grains of rhubar With many, a favorite remedy for the discomfort resulting fro slow digestion is a grain of cayenne pepper, with three or fo grains of rhubarb. The best time for giving medicines for tl purpose in question is just before dinner, and before any oth meal after which a sense of oppression is usually felt.—*Medic Times and Gazette, April* 15, 1854.

